# Latexin expression is downregulated in human gastric carcinomas and exhibits tumor suppressor potential

**DOI:** 10.1186/1471-2407-11-121

**Published:** 2011-04-06

**Authors:** Yong Li, Zhuoma Basang, Huirong Ding, Zheming Lu, Tao Ning, Haoran Wei, Hong Cai, Yang Ke

**Affiliations:** 1 Key Laboratory of Carcinogenesis and Translational Research (Ministry of Education), Department of Genetics, Peking University School of Oncology, Beijing Cancer Hospital & Institute, No. 52 Fucheng Rd, Hai Dian District, Beijing 100142, China; 2 High Altitude Research Center, Medical School, Tibet University, Lhasa, 850002, China

## Abstract

**Background:**

Latexin, also known as endogenous carboxypeptidase inhibitor (CPI), has been found to inhibit mouse stem cell populations and lymphoma cell proliferation, demonstrating its potential role as a tumor suppressor. Our previous study also suggested a correlation between latexin expression and malignant transformation of immortalized human gastric epithelial cells. Here, we examined latexin expression in human gastric carcinomas and investigated the effect of differential latexin expression on proliferation of gastric cancer cells *in vitro *and *in vivo*.

**Methods:**

Monoclonal antibody against human latexin was prepared and immunohistochemical analysis was performed to detect latexin expression in 41 paired gastric carcinomas and adjacent normal control tissues. Human gastric cancer cells MGC803 (latexin negative) stably transfected with LXN gene and BGC823 cells (latexin positive) stably transfected with antisense LXN gene were established for anchorage-dependent colony formation assay and tumorigenesis assay in nude mice. Differentially expressed genes in response to exogeneous latexin expression were screened using microarray analysis and identified by RT-PCR. Bisulfite sequencing was performed to analyze the correlation of the methylation status of LXN promoter with latexin expression in cell lines.

**Results:**

Immunohistochemical analysis showed significantly reduced latexin expression in gastric carcinomas (6/41, 14.6%) compared to control tissues (31/41, 75.6%) (*P *< 0.05). Overexpression of LXN gene in MGC803 cells inhibited colony formation and tumor growth in nude mice. Conversely, BGC823 cells transfected with antisense LXN gene exhibited enhanced tumor growth and colony formation. Additionally, several tumor related genes, including Maspin, WFDC1, SLPI, S100P, and PDGFRB, were shown to be differentially expressed in MGC803 cells in response to latexin expression. Differential expression of Maspin and S100P was also identified in BGC823 cells while latexin expression was downregulated. Further bisulfite sequencing of the LXN gene promoter indicated CpG hypermethylation was correlated with silencing of latexin expression in human cells.

**Conclusions:**

Latexin expression was reduced in human gastric cancers compared with their normal control tissues. The cellular and molecular evidences demonstrated the inhibitory effect of latexin in human gastric cancer cell growth and tumorigenicity. These results strongly suggest the possible involvement of latexin expression in tumor suppression.

## Background

Latexin was originally identified in the lateral neocortex of rats and serves as a marker of regionality and development in rodent nervous systems [1]. Latexin has also been found expressed in various types of human and other vertebrates tissues [2,3]. Human LXN gene [GenBank: NM_020169] encodes latexin protein comprised of 222 amino acids with 84.2% identical to rat, and 84.7% identical to mouse latexin proteins [3]. Human latexin consists of two topologically equivalent subdomains linked by an α-helix and has been found to act as a non-competitive inhibitor of vertebrate carboxypeptidase A and B (CPA and CPB) [4,5]. However, its sequence is unrelated to any other reported carboxypeptidase inhibitor (CPI), but shows significant homology with the putative tumor suppressor, tazarotene-induced gene 1 (TIG1), suggesting a familial relationship [6,7].

Although the biochemical function of latexin as an endogenous CPI has been clearly demonstrated, there are only few reports about the physiological activities of this protein in mammalian cells and the underlying mechanisms remain unclear.

Latexin is the only known inhibitor of CPA in mammals. Human serum CPA activity has been reported to be a potential biomarker for early-stage pancreatic carcinoma, indicating a possible role of latexin in tumorigenesis [8]. Notably, one recent study by Liang and colleagues revealed that latexin functions in the negative control of the hematopoietic stem cell (HSC) populations in mice by decreasing cell replication and increasing apoptosis [9]. Elevated latexin expression has also been reported in normal human stem cells compared to the same cell populations from patients with acute myelogenous leukemia (AML) or lymphoma. The ectopic expression of latexin in mouse lymphoma cells lacking latexin expression show remarkable suppression of growth *in vitro *[10]. Latexin has been suggested to function as a potential tumor suppressor which reduces the risk of old stem cells transforming into cancer stem cells [11].

In a previous study, we identified high levels of latexin expression in an immortalized human gastric epithelium cell line, GES-1 as compared to expression in the MC cell line, which is the malignant derivative of the GES-1 cell line [12]. These findings suggest that downregulation of latexin expression is correlated with malignant transformation of immortalized human gastric epithelial cells. To further investigate latexin expression in human tumors, we collected 41 paired gastric carcinomas and adjacent normal tissues and performed immunohistochemical analysis for latexin expression using an anti-latexin monoclonal antibody. Additionally, human gastric cancer cells with stably increased or decreased latexin expression were established and used to examine the effect of latexin expression on tumor cell growth and tumorigenesis. Moreover, differential expression of genes induced by ectopic latexin expression in cancer cells were screened by microarray analysis, and correlation of LXN promoter methylation with latexin expression was analyzed in human cells.

## Methods

### Cell culture

The GES-1 cell line is an immortalized human gastric epithelial cell which was established by infecting primary fetal gastric epithelial cells with SV40 virus. The MC cell line was derived from GES-1 cells which survived after treatment with the carcinogen N-methyl-N'-nitro-N-nitrosoguanidine (MNNG), and exhibited tumorigenic capability in nude mice [12]. The human gastric adenocarcinoma cell lines MGC803 and BGC823 were obtained from the People's Hospital of Peking University, Beijing, P. R. China. All other cell lines including three immortalized human epithelial cell lines and eight human cancer cell lines were purchased from ATCC (American Type Culture Collection). All cells were maintained in Dulbecco's modified Eagle's medium (DMEM) supplemented with 10% FBS (Gibco, Grand Island, NY), 100 U/ml penicillin and 100 U/ml streptomycin (Invitrogen, Carlsbad, CA) at 37°C in a humidified incubator with 5% CO_2_.

### Preparation of monoclonal antibody against human latexin

Glutathione S-transferase (GST) and GST-latexin fusion proteins were produced by IPTG induction and purified with Glutathione Sepharose 4B (GE Healthcare, Buckinghamshire, UK) according to the manufacturer's instructions. Mouse immunization, cell fusion and HAT selection were carried out as previously described [13]. Three hybridomas were obtained, and one of them, 1G11, was used in this study. 1G11 cells were injected intraperitoneally into BALB/c mice to obtain ascites fluid. Antibody was purified from the ascites fluid with ammonium sulfate precipitation and use of a protein A Sepharose-4B affinity gel (GE Healthcare).

### Cytoplasmic and nuclear protein preparation and Western blot analysis

For each cell line, 2 × 10^6 ^cells were harvested and cytoplasmic and nuclear proteins were extracted using the NE-PER Nuclear and Cytoplasmic Extraction kit (Pierce, Rockford, IL) according to the manufacturer's protocol. For Western blot assay, the cytoplasmic and nuclear proteins were separated by 12% SDS-PAGE and transferred to nitrocellulose membranes (Millipore, Billerica, MA). The membranes were then blocked with 5% nonfat milk and probed with anti-latexin antibody, 1G11 (1:1000 dilution). After washing with 0.2% Tween 20/PBS buffer three times, membranes were incubated with horseradish peroxidase-conjugated anti-mouse IgG (Santa Cruz Biotechnology, Santa Cruz, CA) and visualized using the enhanced chemiluminescence system, ECL (GE Healthcare).

### Tissue samples and immunohistochemical staining

To examine the expression of latexin in human tumor tissues, 41 paired gastric carcinomas and adjacent normal tissue counterparts were used in this study. All tissues were paraffin-embedded and were obtained from the Beijing Cancer Hospital & Institute (Beijing, P.R. China). The slides were deparaffinized and treated with 1% hydrogen dioxide to block endogenous peroxidase. Heat induced epitope retrieval was performed. After preincubating in 0.2% Tween 20/PBS buffer containing 5% dry milk, slides were incubated with anti-latexin monoclonal antibody-1G11 (see description above, 1:100 dilution) at 4°C overnight. After rinsing, biotinylated secondary antibody and horseradish peroxidase labeled streptavidin were added. The signal was developed with DAB-H_2_O_2 _solution, and slides were counterstained with 5% hematoxylin. The brown cytoplasmic signals represent positive staining for latexin. This study was approved by both the Ethics and the Academic committees of Peking University School of Oncology, and informed consent was obtained from each subject.

### Transfection and stable colony selection

Transfection was performed using Lipofectamine 2000 (Invitrogen) based on the manufacturer's instructions. In brief, human gastric cancer cells MGC803 (latexin negative) and BGC823 cells (latexin positive) were seeded in 60 mm-dishes (5 × 10^5 ^cells per dish) and incubated over night. MGC803 cells were then transfected with pcDNA4/TO-LXN or pcDNA4/TO empty vector (Invitrogen), while BGC823 cells transfected with pLXSN-antisense-LXN or pLXSN empty vector (Clontech). Twenty-four hours after transfection, cells were passaged into 10 cm-dishes at 1:20 dilution, and G418 (400 μg/ml, Invitrogen) was added to the growth medium on the following day. The G418 concentration was reduced to 200 μg/ml 5 d after selection, and selection medium was replaced every 3-4 d until individual clones could be identified. After 3 weeks of selection, at least forty G418 resistant clones of MGC803 or BGC823 cells were identified and re-cultured with selective medium. Total protein was extracted and evaluated by Western blot using anti-latexin monoclonal antibody, 1G11. Two clones derived from MGC803 cells stably expressing latexin (C39-8 and C46) and two clones from BGC823 cells with downregulated latexin expression (C3 and C7) were selected for further experiments.

### Colony formation assay

To examine the effect of upregulated or downregulated latexin expression on proliferation of gastric cancer cells, C39-8 and C46 cells with MGC803 cells transfected with pcDNA4/TO empty vector as control, and C3 and C7 cells with BGC823 cells transfected with pLXSN empty vector as control were used for colony formation assay. Each kind of cell was seeded into 6-well plates (1,000 cells/well) and cultured for 2 weeks in medium containing 200 μg/ml G418. These cultures were washed twice with PBS, fixed with a mixture of 25% acetic acid and 75% methanol at room temperature for 15 min, and then stained with 0.4% crystal violet. Clones larger than 2 mm were counted and the number of clones per well was averaged from three wells for each experiment, and three independent experiments were performed.

### Tumorigenicity assay in nude mice

Tumorigenicity assay was performed as previously described [14]. Male BALB/c nude mice (6-7 weeks old) were obtained from the Department of Laboratory Animal Science, Peking University Health Science Center. Cells with upregulated or downregulated latexin expression or control cells were injected subcutaneously into the lateral root of one posterior limb of a nude mouse (1 × 10^6 ^cells/mouse, 8 mice in each experimental group). Tumor size was measured every third day after injection. Tumor volume was calculated according to the formula *V *= (*a *× *b*^2^)/2, where *a *= the largest superficial diameter and *b *= the smallest superficial diameter. Three weeks postinjection, mice were sacrificed and photographed. Care of experimental animals was in accordance with institutional animal care and use committee guidelines.

### Microarray analysis and RT-PCR identification

Total RNA was isolated from C39-8 cells expressing latexin or control MGC803 cells using Trizol reagent (Invitrogen) according to the manufacturer's instructions. Oligonucleotide array analysis was performed by the CapitalBio Corp. (Beijing, P.R. China) using a 22K Human Genome Array (Cat. No. 220010) consisting of 70-mer probes and representing 21,522 human gene transcripts as previously described [15]. Data processing and normalization were performed according to standard procedures using a LOWESS program. The raw microarray data were released into the GEO-database (accession number GSE15787).

To confirm these microarray results, 9 differentially expressed genes were selected for further semi-quantitative reverse transcription-polymerase chain reaction (RT-PCR) testing. Total cellular RNA was reverse transcribed using SuperScript III Reverse Transcriptase (Invitrogen) according to the manufacturer's instructions. The cDNAs obtained were used as template for amplification with HotStarTaq DNA polymerase (Qiagen, Hilden, Germany). For each of the genes examined, two independent amplifications with 2-cycle intervals (N and N+2) were performed to ensure the reliability of RT-PCR results. The primer sets, annealing temperature, cycles for amplification, and length of PCR products are listed in Table 1.

**Table 1:** Primers for RT-PCR analysis of differentially expressed genes induced by latexin expression in MGC803 cells.

Gene symbol	Primers, 5'-3'	Annealing (°C)	Cycles (N)	Product (bp)
*S100P*		59	24	161
F	CGTGGATAAATTGCTCAAGGAC			
R	TCTGCCAGGAATCTGTGACATC			
*PDGFRB*		57	30	605
F	CTCAGGCCACGATGAAAGTG			
R	GTCTCTGTGGACGCAGTTC			
*SLPI*		57	22	239
F	AACCTGAGTGCCAGAGTGAC			
R	AACGCAGGATTTCCCACACA			
*Maspin*		57	23	378
F	AATGCCAAGGTCAAACTCTCC			
R	GCATCCACAGAAAAGTCAGG			
*WFDC1*		59	26	314
F	GCTATGAGTGCCACATCCTGAG			
R	ATCTCTGGGGTCTTGCTCTGCT			
*FBN2*		59	25	561
F	GAATGCTCCAATCCCAATGC			
R	GCTGTCATCGTTCCCTTGAG			
*A2M*		57	22	408
F	CATTGGCTATCTCAACACTGG			
R	TGGTCCCCTTCTTGTGCTGTC			
*BCHE*		59	25	520
F	CATTACACAGACTGGGTAGATG			
R	CTTTCCACTCCCATTCTGCTTC			
*NEP*		57	26	404
F	CTTCGATGACAATGGCAGAAAC			
R	GCAAAGTCCCAATAATCCTGA			
*β-actin*		57	21	638
F	GGAGAAAATCTGGCACCACAC			
R	CGTACAGGTCTTTGCGGATGT			

### Bisulfite sequencing

Cellular genomic DNA was extracted using the standard phenol-chloroform method. Bisulfite treatment of DNA was performed as previously described [16]. Each of bisulfite-modified DNA samples was used as template and a 762 bp fragment covering the entire exon 1 of human LXN gene together with a part of the sequence upstream of exon 1 was amplified with HotStar Taq DNA polymerase (Qiagen) using the following primers: forward, 5'-TAAAAAGTTGAGGATGAGTAAG-3' and reverse 5'-CCTACTAAACTCACCTCCATAC-3'. PCR reactions were performed under the following conditions: an initiation denaturation step at 95°C for 15 min, followed by 34 cycles at 94°C for 30 s, 60°C for 30 s, and 72°C for 50 s, and a final extension step at 72°C for 10 min. The PCR products were then gel-purified and cloned into a pGEM-T Easy Vector (Promega, Madison, WI). Four to 6 individual clones were randomly chosen for sequencing using an ABI PRISM 3730 DNA sequencer (Perkin-Elmer, Foster, CA).

## Results

### Human latexin was expressed predominantly in the cytoplasm

**Figure 1:**
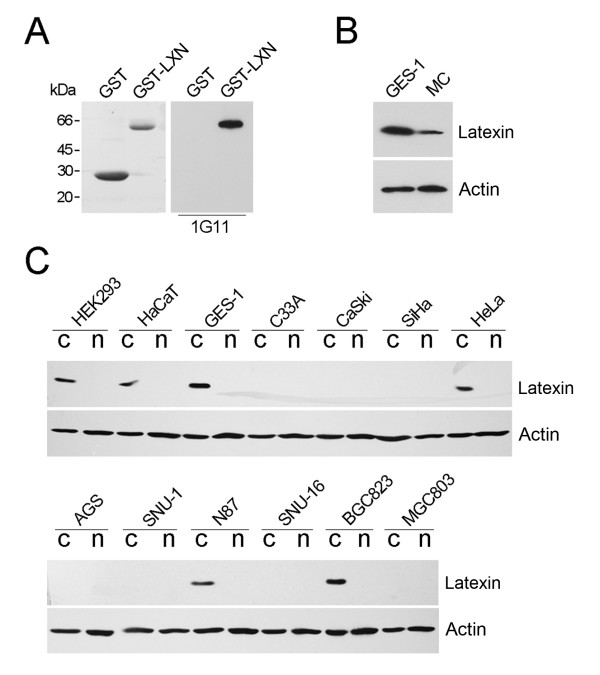
**Analysis of latexin expression in human immortalized and cancerous cells**. (A) Specificity of 1G11, an anti-latexin monoclonal antibody, was tested by Western blot. The left panel is a coomasie stained gel and the right panel shows Western blot with GST and GST-latexin proteins. Expression of latexin (B) in GES-1 and MC cells and (C) in cytoplasmic (c) and nuclear (n) proteins extracted from the indicated cells was identified using Western blot with 1G11 antibody (1:1000 dilution). Actin was used as an internal control.

A monoclonal antibody against human latexin was raised as described in Materials and Methods. To verify the specificity of the anti-latexin monoclonal antibody, 1G11, Western blot analysis was performed with purified GST and GST-latexin fusion proteins. 1G11 specifically recognized GST-latexin but did not bind GST (Figure 1A). The expression of latexin in GES-1 and MC cell lines was then determined by Western blot using 1G11, resulting in the recognition of a 29-kDa protein with a much higher expression level in the GES-1 cells than in the MC cells (Figure 1B). Cytoplasmic and nuclear proteins which were extracted from three immortalized cell lines (HEK293, HaCaT, GES-1) and ten cancer cell lines (cervical cancer cells C33A, CaSki, Hela, and SiHa; gastric cancer cells AGS, SNU-1, N87, SNU-16, BGC823 and MGC803) were evaluated with Western blot. As shown in Figure 1C, the expression of latexin was found predominantly in the cytoplasm of the three immortalized cell lines and in only three of ten cancer cell lines.

### Latexin expression was decreased in human gastric carcinomas compared with normal control tissues

**Figure 2:**
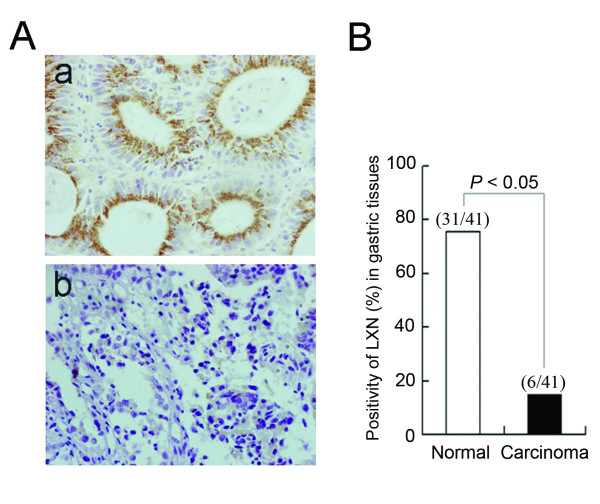
**Analysis of latexin expression in human gastric carcinomas**. (A) Immunohistochemical staining for latexin with monoclonal antibody-1G11 (1:200 dilution). The brown signals represent positive staining for latexin. a, normal gastric tissue; b, gastric carcinoma. Magnification, × 200. (B) The positivity rates of latexin were calculated and illustrated as shown. The number of latexin-positive samples and the total number of each kind of tissue tested here are shown just above each column.

The expression level of latexin was much higher in GES-1 cells than in MC cells, raising the possibility that latexin expression is downregulated in malignant tissue cells. To test this possibility, we investigated latexin expression in 41 gastric carcinomas and paired corresponding adjacent normal tissues by immunohistochemical staining of tissue sections. Positive latexin expression was shown as brown staining in the cell cytoplasm as seen in Figure 2A. Latexin positivity in gastric tissues was calculated and represented Figure 2B. Among the gastric carcinoma tissues tested, only 14.6% (6/41) were latexin positive, while 75.6% of normal gastric tissues (31/41) were latexin positive. These findings suggest a correlation of latexin expression with tumorigenesis of gastric cancer.

### Differential expression of latexin in gastric cancer cells changed the ability of anchorage-dependent colony formation and tumorigenesis in nude mice

**Figure 3:**
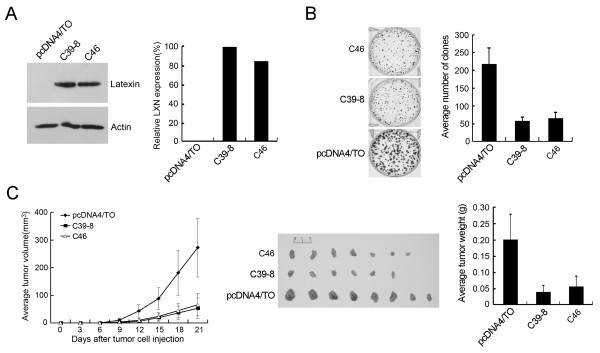
**The effect of ectopic latexin expression on tumor cell growth and tumorigenicity of MGC803 cells**. (A) Latexin expression in gastric cancer cells MGC803 transfected with empty vector (pcDNA4/TO) and MGC803 cells stably overexpressing latexin (C39-8 and C46) was tested by Western blot using 1G11 antibody (1:1000 dilution). (B) C39-8, C46, and control MGC803 cells were used for anchorage-dependent colony formation assay. The photographs show colonies formed by each stable transfectant 2 weeks after plating. Assays were performed in triplicate and the average numbers of clones from one whole well are shown in the graph. (C) C39-8, C46, and control cells were injected subcutaneously into BALB/c nude mice (8 mice/group). The graph (left panel) represents tumor volume as measured on the indicated days. Tumors were excised 21 days after injection (middle panel) and the average weight of tumors of each group was shown as columns (right panel), with bars representing Standard Deviation.

**Figure 4:**
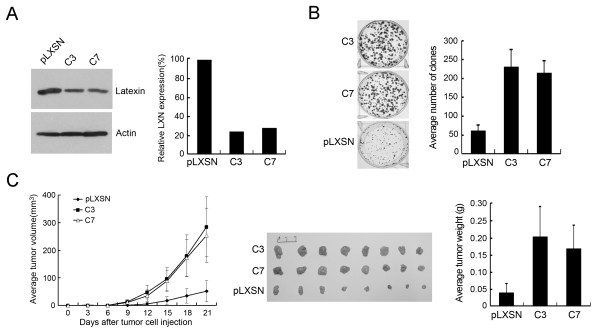
**The effect of downregulated latexin expression on tumor cell growth and tumorigenicity of BGC823 cells**. (A) Gastric cancer cells BGC823 were transfected with an antisense LXN gene expressing vector (pLXSN-antisense-LXN) and two G418-resistant colonies (C3 and C7) with stably decreased latexin expression were selected and tested by Western blot using 1G11 antibody (1:1000 dilution). BGC823 cells transfected with empty pLXSN vector were used as control. (B) C3, C7, and control BGC823 cells were used for colony formation assay. Assays were performed in triplicate and the average numbers of clones were calculated and shown in the graphs. (C) C3, C7, and control BGC823 cells were injected subcutaneously into BALB/c nude mice (8 mice/group). The left graph shows tumor volume as measured on the indicated days. Tumors were excised 21 days after injection (middle panel) and the average weight of tumors of each group was shown as columns (right panel), with bars representing Standard Deviation.

The downregulation of latexin expression in multiple tumor cell lines and tumor tissues suggests that latexin may be functionally involved in the suppression of cancer cell growth. Evaluation of six gastric cancer cell lines showed latexin expression in only two cell lines, BGC823 and N87, but in all other cell lines tested including MGC803 latexin expression was negative (Figure 1C). MGC803 and BGC823 cells were widely used for investigation of tumor cell proliferation, cell-cycle arrest or apoptosis, tumor cell invasion, and the involved cellular mechanisms [17-20]. MGC803 and BGC823 cells were therefore used to evaluate the effect of latexin expression on tumor cell growth. MGC803 cells were transfected with a latexin expressing plasmid (pcDNA4/TO-LXN) and BGC823 cells transfected with an antisense LXN gene expressing plasmid (pLXSN-antisense-LXN), followed by selection of G418-resistant clones. C39-8 and C46 cells with stable overexpression of latexin in MGC803 cells (Figure 3A), and C3 and C7 cells with stably downregulated latexin expression in BGC823 cells (Figure 4A) were obtained and subjected to colony formation assay. As shown in Figure 3B, overexpression of latexin in C39-8 and C46 cells resulted in nearly 70% inhibition of colony formation as compared with MGC803 cells transfected with empty vector (both *P *values < 0.05), and the majority of clones of C39-8 and C46 cells were significantly smaller than those of control cells. Conversely, downregulated latexin expression in C3 and C7 cells caused about 4-fold increase of clones compared to BGC823 cells transfected with empty vector (both *P *values < 0.05, Figure 4B).

The effects of latexin expression on the tumorigenic potential of gastric cancer cells *in vivo *were also evaluated. To this end, MGC803 cells with overexpression of latexin (C39-8 and C46), and BGC823 cells with downregulated latexin expression (C3 and C7) were injected subcutaneously into BALB/c nude mice (8 mice/group). Tumor growth was measured every third day after injection, and after 3 weeks, mice were sacrificed and photographed, and tumors were removed and weighed (Figures 3C and 4C). All 8 nude mice injected with control MGC803 cells developed grossly visible tumors at the site of injection within 3 weeks. In comparison, 6 mice injected with C39-8 cells and 7 mice with C46 cells displayed visible tumors during the same period of time, and both the average tumor weights were significantly less than that of the control group (both *P *values < 0.05). Compared with control BGC823 cells, both C3 and C7 cells showed obviously increased capacity for tumorigenesis (both *P *values < 0.05). Taken together, these results strongly suggest that latexin acts as an inhibitor of tumor cell growth and tumorigenicity.

### Latexin expression induced differential expression of several tumor related genes in human gastric cancer cells

**Figure 5:**
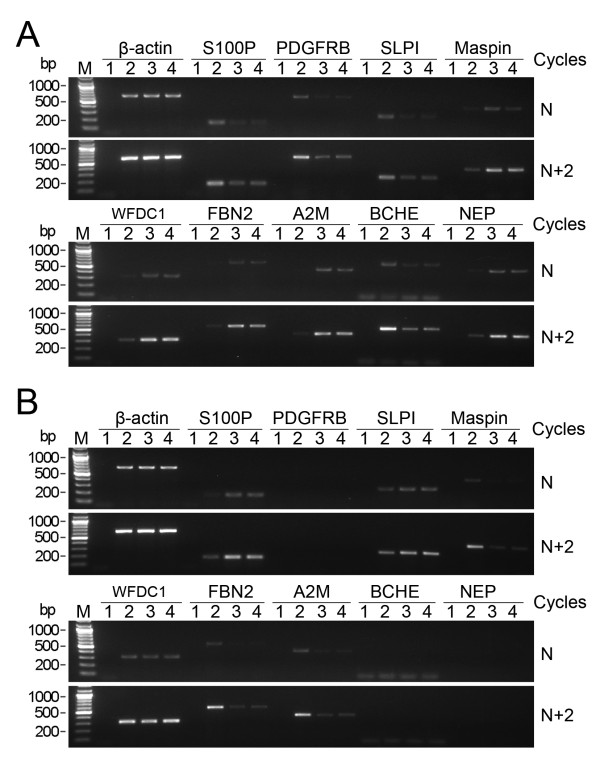
**RT-PCR identification of differential gene expression induced by latexin expression in gastric cancer cells**. To validate the results of Microarray analysis, 9 differentially expressed genes in MGC803 cells in response to latexin expression were subjected to RT-PCR analysis. (A) MGC803 cells stably overexpressing latexin (C39-8 and C46) and control MGC803 cells transfected with empty vector were used for analysis. Lane 1 represents negative control using isolated cellular RNA as PCR template. Lanes 2, 3, and 4 represent amplification using reverse transcripts derived from control MGC803 cells, C39-8, and C46 cells, respectively. (B) The expression of these identified genes was also analyzed in latexin knockdown BGC823 cells, C3 and C7. Lane 1 represents negative control. Lanes 2, 3, and 4 represent amplification results of control BGC803 cells, C3, and C7 cells, separately. For each target gene tested, N and N+2 cycles of RT-PCR amplification were performed to ensure that the PCR reactions fall within the linear range of amplification. The number of cycling (N) for each target gene was shown in Table 1. β-actin was used as the internal control.

To study the molecular mechanism for the inhibitory effect of latexin expression on tumor cell growth, microarray assay was performed to compare gene expression profiles in C39-8 cells and control MGC803 cells transfected with empty vector. The expression of approximately 22,000 genes was tested. Among these, the expression of 18 genes was increased while the expression of another 19 genes was decreased 2 to 11 fold in C39-8 cells in comparison with the control MGC803 cells (Tables 2 and 3). To validate these results, 9 differentially expressed genes were subjected to semi-quantitive RT-PCR analysis. As shown in Figure 5A, four of these, including S100P, PDGFRB, SLPI, and BCHE were found to be downregulated, while 5 genes including Maspin, WFDC1, FBN2, A2M, and NEP were upregulated in C39-8 cells compared with control MGC803 cells. C46 cells were also detected and showed the similar expression changes as C39-8 cells for these 9 genes tested.

**Table 2:** Upregulated genes induced by latexin expression in human gastric cancer cells MGC803.

Gene symbol	Description	RefSeq	Relative fold increase
*A2M*	Alpha 2 macroglobulin	NM_000014	10.3
*NEP*	Neprylisin	NM_007289	5.0
*FBN2*	Fibrillin-2	NM_001999	4.5
*DPT*	Dermatopontin	NM_001937	3.3
*Maspin*	Mammary serine protease inhibitor	NM_021197	3.1
*AKR1C1*	Aldo-keto reductase family 1, member C1	NM_002639	2.9
*WFDC1*	Wap-type four disulfide core 1	NM_001353	2.6
*ABCC3*	ATP-binding cassette, sub-family C	NM_002639	2.5
*HXB*	Hexabrachion	NM_002160	2.5
*SC4MOL*	Sterol-C4-methyl oxidase-like	NM_006745	2.5
*COL7A1*	Collagen, type VII, alpha 1	NM_000094	2.4
*COP*	CARD only protein	NM_052889	2.3
*PEG10*	Paternally expressed 10	XM_496907	2.2
*CSRP2*	Cysteine and glycine-rich protein 2	NM_001321	2.1
*C4BPA*	Complement component 4 binding protein, alpha	NM_000715	2.1
*FLJ23153*	Tumor necrosis-α-induced adipose-related protein	NM_024636	2.1
*PLSCR1*	Phospholipid scramblase 1	NM_021105	2.1
*INSIG1*	Insulin-induced protein 1	NM_198336	2.1

**Table 3:** Downregulated genes induced by latexin expression in human gastric cancer cells MGC803.

Gene symbol	Description	RefSeq	Relative fold decrease
*CGA*	Chorionic gonadotropin-alpha chain	NM_000735	5.9
*FBXO2*	F-box only protein 2	NM_012168	3.7
*MCTP1*	Multiple C2-domains with 2 transmembrane regions 1	NM_024717	3.6
*BCHE*	Butyrylcholinesterase	NM_000055	3.4
*CRABP2*	Retinoic acid-binding protein II, cellular	NM_001878	3.0
*DKK1*	Dickkopf related protein-1 precursor	NM_012242	2.7
*TLR9*	Toll-like receptor 9	NM_017442	2.6
*KIAA1046*	Putative HIV-1 induced protein HIN-1	NM_199324	2.6
*PIR121*	Cytoplasmic FMRP interacting protein 2	NM_014376	2.5
*SLPI*	Secretory leukocyte protease inhibitor	NM_003064	2.3
*TNFAIP8*	Tumor necrosis factor-α-induced protein 8	NM_014350	2.3
*JDP1*	J domain containing protein 1	NM_021800	2.3
*KCNJ2*	Inward rectifier potassium channel 2	NM_000891	2.3
*P2RY6*	P2Y purinoceptor 6	NM_176798	2.3
*S100P*	S100 calcium-binding protein	NM_005980	2.2
*PDGFRB*	Platelet-derived growth factor receptor-beta	NM_002609	2.2
*LCCP*	Leman coiled-coil protein	NM_016201	2.1
*RGC32*	Response gene to complement 32	NM_014059	2.0
*KRT18*	Cytokeratin 18	NM_199187	2.0

Further RT-PCR analysis of C3 and C7 cells revealed that S100P expression was upregulated, and Maspin, FBN2, and A2M expression downregulated by decreased latexin expression in BGC823 cells (Figure 5B). While no obvious expression changes of SLPI and WFDC1, and no expression of PDGFRB, BCHE, and NEP were detected in C3 and C7 cells in comparison with control BGC823 cells.

### CpG methylation regulated transcriptional silencing of LXN gene in human cells

**Figure 6:**
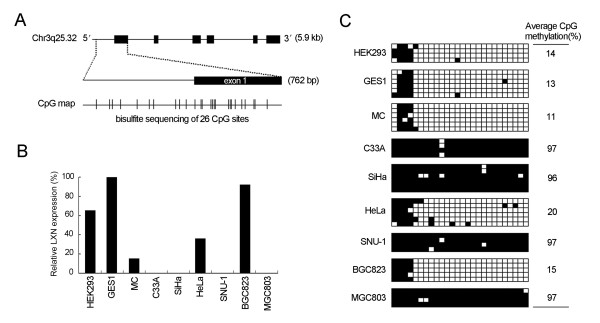
**CpG methylation modifications correlate with LXN gene silencing in human immortalized and cancerous cells**. (A) Schematic diagram of the LXN gene structure derived from the GenBank database. The 26 CpG dinucleotides in exon 1 and the region upstream of exon 1 selected for bisulfite sequencing are indicated in the CpG map. (B) Relative latexin expression in cell lines was evaluated using Western blot normalized to Actin protein level (shown in Figures 1B and 1C). (C) Quantitative measurement of methylation of 26 CpG dinucleotides. Filled and open square boxes indicate methylated and unmethylated CpG sites, respectively. Each row of boxes represents a single clone. Numerical values on the right show the average percentage of methylated CpG sites of each indicated cell line.

The methylation of the LXN gene promoter region was evaluated in various cell lines. A 762 bp fragment which spans exon 1 and the region extending approximately 400 bp upstream of exon 1 of LXN gene [GenBank: NT_005612] is rich in CpG islands, and a total of 26 CpG sites are included in this region as shown in Figure 6A. The sequence analysis of the bisulfite-modified DNA revealed extensive hypermethylation (96-97%) of CpG sites in four cell lines which showed no detectable latexin expression, whereas hypomethylation (11-20%) was detected in five latexin expressing cell lines (Figures 6B and 6C), suggesting that CpG hypermethylation induces transcriptional silencing of LXN gene in human cells. In addition, more than 99% non-CpG cytosines were converted into uracil after bisulfite treatment in this experiment, showing that bisulfite modification of genomic DNA was carried out successfully.

## Discussion

The physiological activity of latexin has been studied relatively little and its effect on cell growth is only now being discovered. In this study, we developed an anti-latexin monoclonal antibody and found that this antibody specifically recognizes a 29-kDa protein, latexin. Western blot analysis of cytoplasmic and nuclear proteins revealed that latexin was located predominantly in the cytoplasm of human cells, and was detected in all three kinds of human immortalized cells and in three of ten human cancer cells tested in this work. Immunohistochemical analysis demonstrated that latexin was expressed in the cytoplasm of tissue cells. Among the gastric carcinoma tissues tested, only 14.6% (6/41) were latexin positive, while 75.6% of normal gastric tissues (31/41) were latexin positive (*P *< 0.05), indicating a correlation of latexin expression with tumorigenesis of gastric cancer. To study the effect of latexin expression on cancer cell growth and tumorigenicity, a LXN gene expression vector was introduced into the latexin-defective human gastric cancer cells MGC803, and two clones (C39-8 and C46) with stable latexin expression were selected and used for colony formation assay and tumor growth assay in nude mice. Both C39-8 and C46 cells exhibited suppressed tumorigenicity and proliferation as compared to control MGC803 cells, suggesting exogenous expression of latexin in MGC803 cells inhibited cancer cell growth. To further examine whether or not the same results will be obtained in other cancer cells, a latexin-positive gastric cancer cell line BGC823 was transfected with an antisense LXN gene expression vector and two clones (C3 and C7) with stably downregulated latexin expression were selected. Inhibition of latexin expression in both C3 and C7 cells accelerated tumor cell growth *in vitro *and in nude mice compared with control BGC823 cells, indicating that decreased latexin expression may contribute to tumorigenesis of gastric carcinoma. As latexin is expressed in normal human tissues of various origins, it is possible that latexin expression may also repress the growth of tumors in other organs.

To investigate the underlying molecular mechanism for the role of latexin expression in negative control of tumor cell growth, we examined the changes in the gene expression profile in response to latexin expression in C39-8 cells compared with MGC803 cells transfected with empty vector. Several genes previously demonstrated to be associated with malignant phenomena were identified by microarray assay and confirmed by RT-PCR. Among these, mammary serine protease inhibitor (Maspin) and wap-type four disulfide core 1 (WFDC1) are tumor suppressor candidates with protease inhibitor activity. Downregulation of Maspin and WFDC1 has been found in a variety of cancers [21-23]. Consistent with its tumor suppressor potential, ectopic expression of latexin in C39-8 cells increased expression levels of Maspin or WFDC1 by more than 2 or 3 fold separately. In contrast, secretory leukocyte protease inhibitor (SLPI) which is also a protease inhibitor but functions to enhance cancer invasion [24] was downregulated by latexin expression to less than one half of control level. In addition, there were two other genes, S100 calcium-binding protein (S100P) and platelet-derived growth factor receptor-beta (PDGFRB), whose expression level was downregulated by latexin expression by more than 2 fold. S100P protein regulates calcium signal transduction and mediates cytoskeletal interaction, protein phosphorylation and transcriptional control. Increased level of S100P expression has also been found to correlate with poor survival in breast and lung cancer, and with progression to metastatic disease in pancreatic cancer [25] and prostate cancer [26]. Platelet-derived growth factors (PDGFs) and their tyrosine kinase receptors (PDGFRA and B) play essential roles in stimulating cell growth and differentiation, and these molecules have been demonstrated to be involved in growth stimulation of tumor cells, tumor angiogenesis and invasiveness [27,28]. Further evaluation of these genes in C46 cells showed the similar expression changes with C39-8 cells, in comparison with control MGC803 cells. Thus, alterations of expression of these genes influenced by latexin expression strongly support the concept that latexin exerts a growth inhibitory function in tumor cells. The expression of these identified genes was also analyzed in C3 and C7 cells which were derived from BGC823 cells by knocking down latexin expression. RT-PCR results showed that S100P expression was upregulated and Maspin expression downregulated by decreased latexin expression in both C3 and C7 cells compared with control BGC823 cells. These results are consistent with the regulatory effect of latexin expression on these two genes found in C39-8 and C46 cells and support the positive effect of downregulated latexin expression on tumor cell growth. Significant expression changes of SLPI, WFDC1, and PDGFRB were observed in MGC803 cells in response to overexpression of latexin, but not in BGC823 cells while latexin expression was downregulated, which may be caused by the relatively limited decrease of latexin expression level in BGC823 cells transfected with antisense LXN gene.

The mechanism by which the LXN gene expression is suppressed in cancer cells has not been demonstrated. It has been established that cytosine hypermethylation of the promoter region results in inactivation of numerous genes in various types of cancers [29,30]. In this study, cytosine methylation status of the LXN gene promoter and the initial exon was examined, and hypermethylation of CpG islands was found to be highly correlated with the transcriptional silencing of LXN gene in nine human cell lines tested. Since downregulated expression of LXN gene was detected in human gastric carcinoma tissues as compared with adjacent normal tissues, the CpG methylation status of the LXN gene in the DNA of paired normal and tumor tissues warrants further investigation.

## Conclusions

We prepared monoclonal antibody against human latexin and found the reduced latexin expression in human gastric carcinomas as compared with normal control tissues. Stable transfection of the LXN gene in human gastric cancer cells MGC803 attenuated cell growth *in vitro *and *in vivo*. In contrast, gastric cancer cells BGC823 transfected with antisense LXN gene expression vector exhibited enhanced capacity for colony formation and tumorigenicity in nude mice. Consistent with its tumor suppressor potential, ectopic expression of latexin induced differential expression of several tumor related genes, including Maspin, WFDC1, SLPI, S100P, and PDGFRB, in gastric cancer cells. In addition, latexin expression in human cells was indicated to be deeply correlated with CpG methylation status of promoter region. Taken together, our results strongly suggest that latexin is a potential tumor suppressor. These findings may open a door for understanding the physiological activity of latexin and related molecular mechanisms in regulation of cell growth.

## Abbreviations

AML: acute myelogenous leukemia; A2M: alpha 2 macroglobulin; BCHE: butyrylcholinesterase; CP: carboxypeptidase; CPI: carboxypeptidase inhibitor; GST: glutathione S-transferase; HSC: hematopoietic stem cell; Maspin: mammary serine protease inhibitor; MNNG: N-methyl-N'-nitro-N-nitrosoguanidine; NEP: neprylisin; PDGFRB: platelet-derived growth factor receptor-beta; RT-PCR: reverse transcription-polymerase chain reaction; SLPI: secretory leukocyte protease inhibitor; S100P: S100 calcium-binding protein; TIG1: tazarotene-induced gene 1; WFDC1: wap-type four disulfide core 1.

## Authors' contributions

YL performed most of the experiments and drafted the manuscript. ZB performed immunohistochemical staining and antisense LXN gene analysis. HD and TN carried out cell transfection and stable colony selection. ZL participated in preparation of 1G11 monoclonal antibody. HW participated in RT-PCR assay. YK and HC performed the data analyses, prepared the manuscript and conceived and supervised this study.

## Pre-publication history

The pre-publication history for this paper can be accessed here:

http://www.biomedcentral.com/1471-2407/11/121/prepub
